# Interventions to prevent or reduce the incidence and severity of ovarian hyperstimulation syndrome: a systematic umbrella review of the best clinical evidence

**DOI:** 10.1186/s12958-023-01113-6

**Published:** 2023-07-21

**Authors:** Stefano Palomba, Flavia Costanzi, Scott M. Nelson, Donatella Caserta, Peter Humaidan

**Affiliations:** 1 Department of Surgical and Medical Sciences and Translational Medicine, Sapienza University of Rome, Sant’Andrea Hospital, via di Grottarossa, n. 1035/1039, Rome, 00189 Italy; 2 School of Medicine, University of Glasgow, Glasgow, UK; 3 NIHR Bristol Biomedical Research Centre, University of Bristol, Oakfield House, Oakfield Grove, Bristol, UK; 4 TFP, Oxford Fertility, Institute of Reproductive Sciences, Oxford, UK; 5 The Fertility Clinic, Faculty of Health, Skive Regional Hospital, Aarhus University, Aarhus C, Denmark

**Keywords:** Assisted reproductive technologies, ART, Complications, In vitro fertilization, Ovarian hyperstimulation syndrome, OHSS, Systematic review

## Abstract

Ovarian hyperstimulation syndrome (OHSS) is a potentially life-threating iatrogenic complication of the early luteal phase and/or early pregnancy after in vitro fertilization (IVF) treatment. The aim of the current study was to identify the most effective methods for preventing of and reducing the incidence and severity of OHSS in IVF patients. A systematic review of systematic reviews of randomized controlled trials (RCTs) with meta-analysis was used to assess each potential intervention (PROSPERO website, CRD 268626) and only studies with the highest quality were included in the qualitative analysis. Primary outcomes included prevention and reduction of OHSS incidence and severity. Secondary outcomes were maternal death, incidence of hospital admission, days of hospitalization, and reproductive outcomes, such as incidence of live-births, clinical pregnancies, pregnancy rate, ongoing pregnancy, miscarriages, and oocytes retrieved. A total of specific interventions related to OHSS were analyzed in 28 systematic reviews of RCTs with meta-analyses. The quality assessment of the included studies was high, moderate, and low for 23, 2, and 3 studies, respectively. The certainty of evidence (CoE) for interventions was reported for 37 specific situations/populations and resulted high, moderate, and low-to-very low for one, 5, and 26 cases, respectively, while it was not reported in 5 cases. Considering the effective interventions without deleterious reproductive effects, GnRH-ant co-treatment (36 RCTs; OR 0.61, 95% C 0.51 to 0.72, *n* = 7,944; I^2^ = 31%) and GnRH agonist triggering (8 RCTs; OR 0.15, 95% CI 0.05 to 0.47, *n* = 989; I^2^ = 42%) emerged as the most effective interventions for preventing OHSS with a moderate CoE, even though elective embryo cryopreservation exhibited a low CoE. Furthermore, the use of mild ovarian stimulation (9 RCTs; RR 0.26, CI 0.14 to 0.49, *n* = 1,925; I^2^ = 0%), and dopaminergic agonists (10 RCTs; OR 0.32, 95% CI 0.23 to 0.44, *n* = 1,202; I^2^ = 13%) coadministration proved effective and safe with a moderate CoE. In conclusion, the current study demonstrates that only a few interventions currently can be considered effective to reduce the incidence of OHSS and its severity with high/moderate CoE despite the numerous published studies on the topic. Further well-designed RCTs are needed, particularly for GnRH-a down-regulated IVF cycles.

## Introduction

Ovarian hyperstimulation syndrome (OHSS) was first described over six decades ago [1] and remains a significant complication associated with ovarian stimulation using gonadotropins, particularly in in vitro fertilization (IVF) cycles, with financial burden [2, 3]. Despite the lack of a formal consensus definition, OHSS is recognized as a potentially life-threatening iatrogenic complication that occurs during the early luteal phase and/or early pregnancy due to an excessive response to ovarian stimulation [4]. Some cases of OHSS cannot be predicted, as they appear to be idiosyncratic reactions to gonadotropins, and spontaneous OHSS cases not related to ovarian stimulation have been reported [5].

OHSS primarily develops when patients with an excessive response to exogenous gonadotropins receive human chorionic gonadotropin (hCG) to complete oocyte maturation, leading to the formation of numerous corpora lutea. The longer half-life of hCG compared to endogenous luteinizing hormone (LH) causes sustained luteotropic activity, inducing vasodilation, increased capillary permeability, and fluid shift from intravascular to extravascular spaces (third space), resulting in hypovolemic hyponatremia [6–8]. Clinically, OHSS is characterized by ovarian cystic enlargement, abdominal distention and pain, and fluid shift from the intravascular space to the third space, potentially leading to ascites, pericardial and pleural effusions, and generalized edema [9]. Life-threatening complications such as, adult respiratory distress syndrome, thromboembolism, and acute renal failure may arise during OHSS [9].

Vascular endothelial growth factors (VEGFs) are key molecules responsible for high vascular permeability [8, 10, 11]. VEGFs are produced by the granulosa cells following gonadotropin stimulation, and their production increases substantially after hCG administration. Additionally, other systemic and local vasoactive substances, including interleukin (IL)-2, IL-6, IL-8, IL-10, IL-18, angiotensin II, histamine, prolactin, prostaglandins, insulin-like growth factor (IGF) 1, and transforming growth factor (TGF) b are also directly and indirectly implicated in OHSS pathogenesis [7, 8, 10, 11]. Genetic predisposition, involving genetic variants of VEGF receptor genes, has also been proposed as a critical factor in OHSS development [10, 12].

The true incidence of the OHSS is challenging to determine due to underreporting [7]. According to the American Society for Reproductive Medicine (ASRM) classification [13], moderate-to-severe OHSS occurs in approximately 1–5% of IVF cycles with an incidence of up to 20% in high-risk patients [7]. Importantly, many OHSS patients seek initial care in the emergency departments. From 2002 to 2011 in the United States (US) there were 11562 hospitalizations due to OHSS and about 4.4% of these cases experienced life-threatening events [9]. A mortality rate of 3/100,000 after IVF cycles due to OHSS was previously estimated in Europe prior to the introduction of the gonadotropin releasing hormone agonist (GnRH-a) trigger protocol [14]. In addition, both in singleton and twin pregnancies, the OHSS is also associated with increased risk of pregnancy complications with a significant incidence of low birth weight and preterm delivery [3].

Various attempts have been made to categorize and classify OHSS [4], with two primary classification modalities described. The first is based on the timing of presentation, distinguishing early and late OHSS forms [15]. The second is based on severity, with numerous classifications proposed in the literature [4]. The most widely used classification delineates OHSS into four stages according to clinical and laboratory features: mild, moderate, severe, and critical forms [16]. However, these grades are not strictly separate and can quickly transition.

A GnRH antagonist (GnRH-ant) cycle followed by a GnRH-a trigger and a “freeze all” policy has proven to be the most effective strategy against OHSS development [17], significantly changing ovarian stimulation and transfer policies worldwide, particularly for women deemed to be at high risk of OHSS. Moreover, following GnRH-a triggering, the risk of early and severe OHSS is not totally cancelled [18]. Due to the effectiveness of the GnRH-a trigger, limited data has subsequently been published on other potential interventions for OHSS prevention/reduction in GnRH-ant co-treated cycles, with conventional hCG triggering or in GnRH-a controlled cycles which are still widely performed globally and in trials exploring new gonadotropin formulations [19–21].

Previous systematic reviews have primarily focused on specific interventions, with clinical guidelines predating recent developments [22] or consensus papers [23], and few attempts were made to summarize the clinical efficacy of many interventions [24]. In light of these shortcomings, we undertook a systematic umbrella review to identify the best evidence-based interventions to prevent or reduce the incidence and severity of OHSS in patients undergoing IVF treatment.

## Methods

This umbrella review was conducted in accordance with the Preferred Reporting Items for Overviews of Reviews (PRIOR) guidelines [25]. The Population, Intervention, Comparison, Outcome (PICO) model [26] guided the study design. The review protocol (CRD 268626) was registered on the PROSPERO website (http://www.crd.york.ac.uk/PROSPERO).

### Review question

The primary question was: Which interventions are most effective, based on the best clinical evidence, for preventing and reducing the incidence and severity of OHSS in patients undergoing IVF?

### PICO model

In accordance with the PICO model [26], the “Population” comprised infertile patients undergoing IVF and/or intracytoplasmic sperm injection (ICSI) treatment. The “Intervention” encompassed each strategy, procedure, or treatment employed before, during or after ovarian stimulation that potentially affects OHSS risk and severity. The “Comparison” involved no intervention or a placebo/sham arm or another potentially active intervention. Primary and secondary “Outcomes” were ranked by importance in evaluating intervention effects. Incidence and severity of OHSS were considered primary (critical) outcomes. Secondary outcomes included maternal death (critical), incidence of hospital admission (critical), days of hospitalization (important), live birth rate (critical), clinical pregnancy rate (critical), pregnancy rate (important), ongoing pregnancy rate (important), miscarriage rate (important), and number of oocytes retrieved (important).

### Data sources and search strategy

An initial search was conducted in November 2022 using the keywords “OVARIAN HYPERSTIMULATION SYNDROME” and “OHSS” in PubMed, The Cochrane Library and Web of Science. The literature search aimed to identify all potential interventions that assessed the incidence and/or severity of OHSS. A subsequent formal search was performed, pairing each specific intervention identified with “OVARIAN HYPERSTIMULATION SYNDROME” or “OHSS” to detect all interventions analyzed in systematic reviews.

### Eligibility criteria

Inclusion criteria encompassed human studies published in English. No publication period restrictions were applied. For the first search, no additional specific inclusion and exclusion criteria were considered. During the second literature search for each identified intervention, only systematic reviews of randomized controlled trials (RCTs) with meta-analyses with data related to OHSS were included in the final analysis. Systematic reviews were defined as studies that collect data from primary research studies using organized, repeatable procedures and subsequently synthesize the quantitative or qualitative results. Studies with different designs, including network meta-analyses [27, 28] were excluded.

If two or more studies were available, the inclusion criteria prioritized the highest quality study, followed by the most recent study. Overlapping systematic reviews were included only if they had similar quality and were published in the same year or if the selected study did not report important sub-analyses. No additional searches for supplemental primary studies were performed, and unpublished studies were not specifically sought. The authors also hand-searched the reference lists of included articles and previous reviews to find additional data relevant to the of interest to the study’s aim. Searches were re-run prior to the final analysis.

### Data collection process

Two authors (SP, FC) performed, extracted, and tabulated all searches with three others (DC, PH, SMN) checking the results. For each specific intervention, a custom table to extract data was created to extract data. Data extracted and tabulated included the first author, year of publication, country, study design (systematic reviews and supplemental primary RCTs), population characteristics, studies included, sample size, ovarian stimulation protocols, primary and secondary outcomes (as detailed earlier), and the certainty of evidence (CoE). No attempts were made to obtain original data by contacting corresponding authors.

### Quality assessment

Two authors (SP, FC) assessed the quality of all included studies. The Assessing the Methodological Quality of Systematic Reviews 2 (AMSTAR-2; http://www.amstar.ca) [29] was used for systematic review evaluation (Table 1).

**Table 1:** Quality assessment of systematic reviews according to AMSTAR-2 [29]

**HIGH**	**No or one non-critical weakness**	The systematic review provides an accurate and comprehensive summary of the results of the available studies that address the question of interest
**MODERATE**	More than one non-critical weakness*	The systematic review has more than one weakness but no critical flaws. It may provide an accurate summary of the results of the available studies that were included in the review
**LOW**	**One critical flaw with or without non-critical weaknesses**	The review has a critical flaw and may not provide an accurate and comprehensive summary of the available studies that address the question of interest
**CRITICALLY LOW**	**More than one critical flaw with or without non-critical weaknesses**	The review has more than one critical flaw and should not be relied on to provide an accurate and comprehensive summary of the available studies
**Critical domains** • Protocol registered before commencement of the review (item 2) • Adequacy of the literature search (item 4) • Justification for excluding individual studies (item 7) • Risk of bias from individual studies being included in the review (item 9) • Appropriateness of meta-analytical methods (item 11) • Consideration of risk of bias when interpreting the results of the review (item 15)		
**Non-critical weakness** • PICO model (item 1) • Explain the selection for the inclusion (item 3) • Selection of studies in duplicate (item 5) • Data extraction in duplicate (item 6) • Describe the included studies (item 8) • Funding sources for the studies included in the review (item 10) • Potential impact of risk of bias in individual studies on outcomes (item 12) • Consideration of the risk of bias in individual studies when interpreting/discussing the results (item 13) • Heterogeneity observed (item 14) • Conflict of Interest (item 16)		

*Multiple non-critical weaknesses may diminish confidence in the review, and it may be appropriate to move the overall appraisal down from moderate to low confidence

### Data analysis

A qualitative analysis was performed for each intervention, alone or in combination. Quantitative analysis, using aggregate data, was reported as detailed in the original papers. Similarly, the CoE regarding the intervention effect on OHSS risk/severity and data heterogeneity (inconsistency measure, I^2^) [30] were reported as detailed in the original meta-analysis papers. The CoE was reported for each specific intervention (for example GnRH-ant for general, unselected, PCOS, and poor-responder population).

Data were also sub-analyzed according to use of GnRH-a or GnRH-ant for pituitary down-regulation, to hCG or GnRH-a for ovulation trigger, and to different populations (unselected, PCOS, and so on).

### Ethics

No formal ethical approval was required as the study did not involve humans or the use of human tissue or hospital records samples, and no personal data were recorded and analyzed.

## Results

**Fig. 1:**
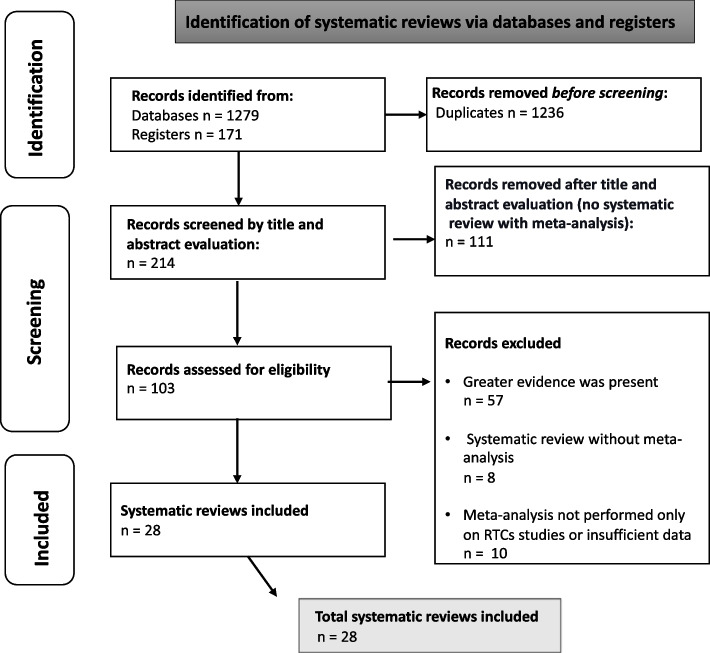
PRIOR flow diagram [25]

In our initial search, 8,976 items were identified and assessed through abstract and full-text examination as necessary. This led to the identification of 46 potential interventions. Following the second literature search, 1,450 records were obtained, with 1,236 being excluded due to duplication. Of the remaining 214 records, 103 were chosen for eligibility assessment after title and abstract evaluation. Subsequently, 76 out of 103 records were excluded for the following reasons: 57 had superior evidence available, 8 lacked data synthesis, and 10 featured meta-analyses that included non-RCTs or insufficient data. Ultimately, 28 studies representing 37 interventions were included in this umbrella review (Fig. 1). Table 2 presents all intercepted interventions with potential effects on OHSS risk analyzed or did not analyze in systematic reviews with meta-analyses of RCTs. Table 3 outlines the main characteristics of the studies included in the final analysis.

**Table 2:** All interventions identified to potentially modify OHSS risk

Interventions	Systematic review with meta-analysis
Alternative hCG protocol	✖
Aspirin	✖
Calcium infusion	✔
Carbegoline	✔
Clomiphene citrate	✔
Coasting	✔
Corifollitropin alfa	✔
Cycle cancellation	✖
Diosmin	✔
Dopaminergic agonists	✔
Dual trigger	✖
Elective cryopreservation	✔
Elective single embryo transfer (e-SET)	✖
Follitropin delta	✖
FSH dose decrease	✖
Glucocorticoid	✔
GnRH analogs	✔
Inositol	✖
Insulin sensitizing drugs	✖
In vitro maturation of oocytes	✔
Intensified luteal phase support with hCG	✖
Intensified luteal phase support: GnRH agonist	✔
Ketoconazole	✖
Kisspeptin	✖
Letrozole	✔
LH addition	✔
Luteal GnRH antagonist administration	✖
Luteal phase support / GnRH agonist	✔
Luteal phase support / hCG	✔
Luteal phase support / progesterone	✔
Melatonin	✔
Metformin	✔
Mild ovarian stimulation	✔
Monitoring and surveillance	✔
Natural IVF cycles	✔
Oral contraceptives	✔
Ovarian drilling	✔
Personalization	✔
Predictive models	✔
Progestin-primed ovarian stimulation	✔
Triggering / hCG dose	✖
Triggering / GnRH agonist	✔
Triggering / r-hLH	✔
Triggering / hCG type	✔
Gonadotropins	✔
Vitamin D	✖
Volume expanders / albumin	✔
Volume expanders / hydroxyethyl starch	✔

**Table 3:** Characteristics of the studies included in the final analysis according to the specific intervention

Interventions	Evidence	Country	Ovarian stimulation protocol	Population	CoE	Assessment of quality ^a^
**Predictive models**	Lensen et al., 2018	New Zealand	4 RCTs: 2 agonists; 1 antagonists; 1 no OS performed	General infertile population	Low	High
**Monitoring and surveillance**	Kwan et al., 2021	England	6 RCTs: 3 agonists; 1 both agonists and antagonists; 1 hMG alone; 1 unknown	General infertile population	Low	High
**Natural IVF cycles**	Allersma et al., 2013	Netherlands	1 RCT: Natural IVF cycles vs. agonist	General infertile population	Very low	High
**Pre-treatment with oral contraceptives**	Farquhar et al., 2017	New Zealand	2 RCTs: antagonists vs. pre-treatment with OC + antagonists 2 RCTs: pre-treatment with OC + agonists	General infertile population	Low	High
**Personalization**	Lensen et al., 2018	New Zealand	1 RCT: agonists OR agonists + 100 UI vs. 150 UI FSH	General infertile population	Very low	High
**Mild ovarian stimulation**	Datta et al., 2021	England	Normal-responder 9 RCTs: 6 antagonists vs. agonists; 2 agonists; 1 no treatment vs. agonists Hyper-responder 2 RCTs: 1 antagonists vs. agonists; 1 agonists or antagonists	General infertile population	Moderate	High
**Urinary vs. recombinant gonadotropin**	van Wely et al., 2011	Netherlands	32 RCTs: 29 agonists; 1 antagonists, 2 unknown	General infertile population	High	High
**Corifollitropin alfa**	Cozzolino et al., 2018	Spain	5 RCTs: antagonists	General infertile population	Not available	Moderate
**r-LH**	Mochtar et al., 2017	England	6 RCTs: 4 agonists, 2 antagonists	General infertile population	Low	High
**Clomiphene citrate or letrozole**	Kamath et al., 2017	India	5 RCTs: 2 agonists; 2 agonists vs. antagonists; 1 antagonists	General infertile population	Low	High
**Clomiphene citrate**	Bechtejew et al., 2017	Brazil	4 RTCs: unknown	General infertile population	Moderate	Moderate
**Letrozole**	Bechtejew et al., 2017	China	1 RTC: unknown	General infertile population	Low	Moderate
**Metformin**	Tso et al., 2020	Brazil	11 RCTs: 9 agonists; 2 antagonists	PCOS Patients	Low	High
**Melatonin**	Seko et al., 2014	Brazil	1 RCT: agonists	PCOS	Low	High
**Coasting**	D’Angelo et al., 2017	England	2 RCTs: coasting vs. no coasting	General infertile population	Low	High
**GnRH analogs** **Unselected population**	Al-Inany et al., 2016	Egypt	36 RCTs: antagonists vs. agonists	General infertile population	Moderate	High
**GnRH analogs** **General population**	Lambalk et al., 2017	Netherlands	22 RCTs and 9 RCTs: antagonists vs. agonists	General infertile population and PCOS patients	Not available	High
**GnRH analogs** **PCOS population**	Kadoura et al., 2022	Syrian Arab Republic	9 RCTs: antagonists vs. agonists	PCOS	Very low	High
**GnRH analogs** **Normo-responders**	Wang et al., 2017	China	21 RCTs: antagonists vs. agonists	Normal responders	Not available	High
**GnRH analogs** **Poor-responders**	Lambalk et al., 2017	Netherlands	6 RCTs: antagonists vs. agonists	Poor-responders	Not available	High
**Progestin-primed ovarian stimulation**	Guan et al., 2021	China	6 RCTs: 5 antagonists, 1 agonists	General infertile population	Low	Low
**Triggering: type of hCG**	Youssef et al., 2016a	Egypt	3 RCTs: 2 agonists, 1 antagonists	General infertile population	Low	High
**Triggering: GnRH agonist**	Youssef et al., 2014	Egypt	8 RCTs: 8 antagonists	General infertile population	Moderate	High
**Triggering: r-hLH**	Youssef et al., 2016a	Egypt	2 RCTs: 2 agonists	General infertile population	Very low	High
**Elective cryopreservation**	Zaat et al., 2021	Netherlands	6 RCTs: 3 antagonists, 2 agonists, 1 no GnRH analogs	General infertile population	Low	High
**In vitro maturation of oocytes**	Siristatidis et al., 2018	Greece	11 prospective and retrospective studies. No cases of OHSS were reported	Patients with and without PCOS	Very low	Low
**Dopaminergic agonists**	Tang et al., 2021	Netherlands	10 RCT: unknown	General infertile population	Moderate	High
**Diosmin**	Tang et al., 2021	China	1 RCT: unknown	General infertile population	Very low	High
**Volume expanders: albumin**	Youssef et al., 2016b	Egypt	7 RCTs: 6 agonists; 1 unknown	General infertile population	Very low	High
**Volume expanders: hydroxyethyl starch**	Youssef et al., 2016b	Egypt	2 RCTs: 2 agonists	General infertile population	Very low	High
**Glucocorticoid**	Boomsma et al., 2022	Netherlands	3 RCTs: 3 agonists	General infertile population	Very low	High
**Luteal phase support: hCG**	Van der Linden et al., 2015	Netherlands	1 RCT: agonists	General infertile population	Low	High
**Luteal phase support: progesterone**	Van der Linden et al., 2015	Netherlands	5 RCTs: 5 agonists	General infertile population	Low	High
**Luteal phase support: GnRH agonist** **(*vs.* progesterone)**	van der Linden et al., 2015	Netherlands	1 RCT: agonists	General infertile population	Very low	High
**Intensified luteal phase support: GnRH agonist**	Ma et al., 2019	China	2 RCTs: 1 agonists; 1 antagonists	General infertile population	Not available	Low
**Calcium infusion**	Tang et al., 2021	China	2 RCTs: 2 agonists	General infertile population	Very low	High
**Ovarian drilling**	Bordewijk *et al.,* 2020	Netherlands	1 RCT: agonists	Infertile population with PCOS	Very low	High

*CC* Clomiphene citrate, *CoE* Certainty of evidence, *FSH* Follicle-stimulating hormone, *hMG* Human menopausal gonadotrophin, *IVF* In vitro fertilization, *LH* Luteinizing hormone, *OC* Oral contraceptives, *PCOS* Polycystic ovary syndrome, *SR* Systematic review, *UI* International unit

^a^Assessing the Methodological Quality of Systematic Reviews 2 (AMSTAR-2, http://www.amstar.ca) [29]

For each intervention analyzed, we provide the rationale for its use, available/intercepted studies (if more than one and avoiding citing papers subsequently updated), primary outcomes, CoE, and study quality. Table 4 summarizes the primary and secondary outcomes for each intervention. The quality assessment for the 28 included systematic reviews of RCTs with meta-analysis was deemed high, moderate, and low for 23, 2, and 3 studies, respectively. We assessed the CoE for the effect on OHSS risk of the interventions intercepted on specific populations or clinical situations (a total of 37 items) resulting high, moderate, and low to very low for one, 5, and 26 cases, respectively. Five interventions lacked reported CoE (Table 4).

**Table 4:** Primary and secondary endpoints for each specific intervention according to different population/ clinical situations

	Total OHSS	Moderate-severe OHSS	Live-births	Clinical pregnancies	Ongoing pregnancy rate	Pregnancy rate	Miscarriages	Oocytes retrieved
**Predictive models**	**No difference** 4 RCTs; OR 0.58, 95% CI 0.34 to 1.00, *n* = 2823; I^2^ = 0%	/	**No difference** 4 RCTs; OR 1.04, 95% CI 0.88 to 1.23, *n* = 2823; I^2^ = 34%	**No difference** 4 RCTs; OR 0.96, 95% CI 0.82 to 1.13, *n* = 2823; I^2^ = 0%	**No difference** 4 RCTs; OR 1.04, 95% CI 0.88 to 1.23, *n* = 2823; I^2^ = 34%	/	/	/
**Monitoring and surveillance**	**No difference** 6 RCTs; OR 1.03, 95% CI 0.48 to 2.20, *n* = 781; I^2^ = 0%	/	/	/	/	**No difference** 4 RCTs; OR 1.10, 95% CI 0.79 to 1.54, *n* = 617; I^2^ = 5%	/	**No difference** 4 RCTs; OR 0.32, 95% CI -0.60 to 1.24; *n* = 596; I^2^ = 17%
**Natural IVF cycles**	**No difference** 1 RCT; OR 0.19, 95% CI 0.01 to 4.06, *n* = 60	/	/	**No difference** 4 RCTs; OR 0.52, 95% CI 0.17 to 1.61, *n* = 351; I^2^ = 63%	**No difference** 3 RCTs; OR 0.72, 95% CI 0.50 to 1.05, *n* = 458; I^2^ = 0%	/	/	**Reduction** 1 RCT; MD -4.40, 95% CI -7.87 to -0.93, *n* = 60
**Pre-treatment with oral contraceptives + GnRH analogues**								
***vs.*** **GnRH antagonists**	**No difference** 2 RCTs; OR 0.98, 95% CI 0.28 to 3.40, *n* = 642; I^2^ = 0%	/	**Reduction** 6 RCTs; OR 0.74, 95% CI 0.58 to 0.95, *n* = 1335; I^2^ = 0%	/	**Reduction** 6 RCTs; OR 0.74, 95% CI 0.58 to 0.95, *n* = 1335; I^2^ = 0%	/	**No difference** 5 RCTs; OR 1.36, 95% CI 0.82 to 2.26, *n* = 868; I^2^ = 0%	/
***vs.*** **GnRH agonists**	**No difference** 2 RCTs; OR 0.63, 95% CI 0.20 to 1.96, *n* = 290; I^2^ = 0%	/	**No difference** 4 RCTs; OR 0.89, 95% CI 0.64 to 1.25, *n* = 724; I^2^ = 0%	/	**No difference** 4 RCTs; OR 0.89, 95% CI 0.64 to 1.25, *n* = 724; I^2^ = 0%	/	**Reduction** 5 RCTs; OR 0.40, 95% CI 0.22 to 0.72, *n* = 780; I^2^ = 0%	/
**GONADOTROPIN STARTING DOSE**								
**Personalization** (In high-responders)	/	**Reduction** 1 RCT; OR 2.31, 95% CI 0.80 to 6.67, *n* = 521;	**No difference** 1 RCT; OR 0.98, 95% CI 0.66 to 1.46, *n* = 521	**No difference** 1 RCT; OR 1.14, 95% CI 0.78 to 1.66, *n* = 521	/	/	/	/
** Mild ovarian stimulation**								
**Normal-responders**	**Reduction** 9 RTCs; RR 0.26, CI 0.14 to 0.49, *n* = 1925; I^2^ = 0%	**/**	**No difference** 3 RCTs; OR 0.88, 95% CI 0.69 to 1.12, *n* = 573; I^2^ = 0%	**/**	**No difference** 7 RCTs; OR 1.10, 95% CI 0.88 to 1.12, *n* = 573; I^2^ = 0%	**/**	**/**	**Reduction** 13 RTCs; SMD-1.34, CI -1.94 to -0.75, *n* = 3499; I^2^ = 98%
**Hyper-responders**	**Reduction** 2 RTCs; RR 0.47, 95% CI 0.31 to 0.72, *n* = 931; I^2^ = 0%	**/**	**No difference** 2 RCTs; OR 0.98, 95% CI 0.79 to 1.22, *n* = 931; I^2^ = 0%	**/**	**No difference** 1 RCT; OR 0.86, 95% CI 0.61 to 1.23, *n* = 521;	**/**	**/**	**No difference** 2 RTCs; SMD-0.31, CI -0.74 to -0.13, *n* = 931, I^2^ = 91%
**PROTOCOL OF INDUCTION: GNRH AGONISTS *VS. *ANTAGONIST**								
** DRUG FORMULATION FOR OVARIAN STIMULATION**								
**Urinary vs. recombinant gonadotropin**	**No difference** 32 RCTs; OR 1.18, 95% CI 0.86 to 1.61, *n* = 7740; I^2^ = 0%	**/**	**No difference** 28 RCTs; OR 0.97, 95% CI 0.87 to 1.08, *n* = 7339; I^2^ = 0%	**/**	**No difference** 28 RCTs; OR 0.97, 95% CI 0.87 to 1.08, *n* = 7339; I^2^ = 0%	**/**	**/**	**/**
**Corifollitropin alfa** (including poor- and normal-responders)	**No difference** 5 RCTs; RR 1.15, 95% CI 0.83 to 1.57, *n* = 3749; I^2^ = 0%	**No difference** 4 RCTs; RR 1.17, 95% CI 0.54 to 2.56, *n* = 3349; I^2^ = 0%	**No difference** 8 RCTs; RR 0.92, 95% CI 0.80–1.05, *n* = 4340; I^2^ = 23%	**No difference** 7 RCTs; RR 0.96, 95% CI 0.88 to 1.05, *n* = 4340; I^2^ = 0%	**No difference** 8 RCTs; RR 0.92, 95% CI 0.80–1.05, *n* = 4242; I^2^ = 23%	/	**No difference** 4 RCTs; RR, 0.94; 95% CI, 0.71–1.25, *n* = 4242; I^2^ = 0%	/
** REGIMENS OF OVARIAN STIMULATION**								
**Clomiphene citrate or** **letrozole**	**Reduction** 5 RCTs; OR 0.21, 95% CI 0.11 to 0.41, *n* = 1067; I^2^ = 0%	**/**	**No difference** 4 RCTs; RR 0.92, 95% CI 0.66 to 1.27, *n* = 493; I^2^ = 0%	**No difference** 12 RCTs; RR 1.00, 95% CI 0.86 to 1.16, *n* = 1998; I^2^ = 3%	**/**	**/**	**/**	**/**
**Clomiphene citrate**	**Reduction** 4 RTCs; OR 0.2, 95% CI, 0.1 to 0.3, *n* = 1251; I^2^ = 0%	/	**No difference** 4 RCTs; RR 0.9, 95% CI 0.7 to 1.1, *n* = 1207; I^2^ = 36%	**No difference** 8 RCTs; RR 1.0, 95% CI 0.9 to 1.1, *n* = 1764; I^2^ = 0%	/	/	**No difference** 4 RCTs; RR 0.83, 95% 0.59 to 1.19, *n* = 610	**Reduction** 8 RCTs; MD − 4.6, 95% CI − 6.1 to − 3, *n* = 1631; I^2^ = 95%
**Letrozole**	**Reduction** 1 RTC; OR 0.1, 95% CI, 0.0 to 0.6 *n* = 94	/	**/**	**/**	**/**	**/**	**/**	**/**
**Metformin** **GnRH agonists** **GnRH antagonists**	**Reduction** 9 RCTs; RR 0.40, 95% CI 0.26 to 0.60, *n* = 898; I^2^ = 23% **No difference** 2 RCTs; RR 0.97, 95% CI 0.32 to 2.98, *n* = 193; I^2^ = 25%	/ /	**No difference** 6 RCTs; RR 1.30, 95% CI 0.94 to 1.79, *n* = 651; I^2^ = 23% **Reduction** 1 RCT; RR 0.48, 95% CI 0.29 to 0.79, *n* = 153	**Increase** 10 RCT; RR 1.32, 95% CI 1.08 to 1.63, *n* = 915; I^2^ = 13% **No difference** 2 RCTs; RR 1.38, 95% CI 0.21 to 9.19, *n* = 177; I^2^ = 87%	/ /	/ /	**No difference** 7 RCTs; RR 0.80, 95% CI 0.51 to 1.26, *n* = 668; I^2^ = 0% **No difference** 1 RCT; RR 0.86, 95% CI 0.56 to 1.32, *n* = 153	/ /
**Melatonin**	**Reduction** 1 RCT; RR 1.01; 95% CI 0.33 to 3.08, *n* = 358	/	**/**	**No difference** 5 RCTs; RR 0.6; 95% CI 0.2–2.22, *n* = 680; l^2^ = 0%	**/**	**/**	**No difference** 2 RCTs; RR 1.7; 95% CI 0.43–2.68, *n* = 143; l^2^ = 0%	**No difference** 5 RCTs; MD 0.6; -02 to -1.4, *n* = 680; I^2^ = 69%
**Coasting**	**Reduction** 2 RCTs; OR 0.11, 95% CI 0.05 to 0.24, *n* = 207; I^2^ = 0%	**/**	**No difference** 1 RCT; OR 0.48, 95% CI 0.14 to 1.62, *n* = 68	**No difference** 2 RCTs; OR 0.82, 95% CI 0.46 to 1.44, *n* = 207; I^2^ = 0%	**/**	**/**	**No difference** 2 RCTs; OR 0.85, 95% CI 0.25 to 2.86, *n* = 207, I^2^ = 0%	/
** STRATEGIES FOR CONTROLLING LH SURGE**								
**GnRH agonist *vs.*** **antagonists: overall population**	**Reduction in antagonists** 36 RCTs; OR 0.61, 95% C 0.51 to 0.72, *n* = 7944; I^2^ = 31%	**/**	**No difference** 12 RCTs; OR 1.02, 95% CI 0.85 to 1.23, *n* = 2303; I^2^ = 27%	**No difference** 54 RCT; OR 0.91, 95% CI 0.83 to 1, *n* = 9959; I^2^ = 31%	**No difference** 37 RCT; OR 0.92, 95% CI 0.83 to 1.01, *n* = 8311; I^2^ = 0%	**/**	**No difference** 34 RCTs; OR 1.03, 95% CI 0.82 to 1.29, *n* = 7082; I^2^ = 0%	**/**
**GnRH agonists *vs.*** **antagonists: general population**	**Reduction in antagonists** 22 RCTs; OR 0.63, 95% C 0.50 to 0.81, *n* = 5598; I^2^ = 0%	**/**	**No difference** 10 RCTs; OR 0.91, 95% CI 0.79 to 1.04, *n* = 2590; I^2^ = 0%	**No difference** 34 RCT; OR 0.90, 95% CI 0.84 to 0.96, *n* = 8084; I^2^ = 0%	**Reduction** 26 RCT; OR 0.89, 95% CI 0.82 to 96, *n* = 7191; I^2^ = 0%	**/**	**No difference** 34 RCTs; OR 1.03, 95% CI 0.82 to 1.29, *n* = 7082; I^2^ = 0%	**Increase in agonists** 31 RCTs; WMD − 1.04, CI -1.56 to -0.52, *n* = 7080 I^2^ = 81%
**GnRH agonists *vs.*** **antagonists: PCOS**	**Reduction in antagonists** 9 RCTs; OR 0.58, 95% C 0.44 to 0.77, *n* = 994, I^2^ = 0%	**Reduction in antagonist** 9 RCTs; OR 0.65, 95% C 0.52 to 0.82, *n* = 1114, I^2^ = 0%	**No difference** 1 RCT; OR 0.78, 95% CI 0.46 to 1.32, *n* = 74	**No difference** 8 RCTs; OR 0.96, 95% CI 0.77 to 1.19, *n* = 840; I^2^ = 30%	**No difference** 5 RCTs; OR 0.92, 95% CI 0.78 to 1.08, *n* = 785; I^2^ = 0%	**/**	**No difference** 7 RCTs; OR 0.93, 95% CI 0.61 to 1.43, *n* = 997; I^2^ = 1%	**/**
**GnRH agonists *vs.*** **antagonists: normal responder**	**Reduction in antagonists** 21 RCTs; OR 0.69, 95% CI 0.57 to 0.83 *n* = 5763; I^2^ = 15%	**/**	**No difference** 6 RCTs; OR 0.95, 95% CI 0.74 to 1.09, *n* = 2237; I^2^ = 0%	**No difference** 8 RCTs; OR 0.90, 95% CI 0.80 to 1.01, *n* = 5814; I^2^ = 0%	**No difference** 18 RCTs; OR 0.88, 95% CI 0.77 to 1.00, *n* = 5119; I^2^ = 0%	**/**	**No difference** 14 RCTs; OR 0.98, 95% CI 0.69 to 1.40, *n* = 3198; I^2^ = 0%	**Reduction in antagonists** 22 RTCs; SMD-1.14, CI -1.84 to -0.99, *n* = 4914, I^2^ = 45%
**GnRH agonists *vs.*** **antagonists: poor responder**	**/**	**/**	**No difference** 3 RCTs; OR 0.89, 95% CI 0.56 to 1.41, *n* = 544; I^2^ = 0%	**No difference** 6 RCTs; OR 0.85, 95% CI 0.66 to 1.10, *n* = 780; I^2^ = 0%	**No difference** 6 RCTs; OR 0.87, 95% CI 0.65 to 1.17, *n* = 780; I^2^ = 0%	**/**	**/**	**Increase in agonist** 6 RCTs; WMD − 0.08, CI -1.09 to -0.43, *n* = 7080 I^2^ = 57%
**Progestin-primed ovarian stimulation**	**Reduction** 6 RCT; OR 0.52, 95% CI 0.36 to 0.75, *n* = 1238; I^2^ = 0%	/	**No difference** 6 RCTs; RR 1.06, 95% CI 0.94 to 1.19, *n* = 1442; I^2^ = 0%	**No difference** 8 RCTs; RR 0.99, 95% CI 0.85 to 1.15, *n* = 1782; I^2^ = 56%	**No difference** 6 RCTs; RR 1.06, 95% CI 0.94 to 1.19, *n* = 1442; I^2^ = 0%	/	**No difference** 8 RCTs; RR -0.03, 95% CI -0.35 to 0.29, *n*=; I^2^ = 0%	**/**
**LH addition**	**No difference** 6 RCTs; OR 0.38, 95% CI 0.14 to 1.01, *n* = 2178; I^2^ = 10	/	**No difference** 4 RCTs OR 1.32, 95% CI 0.85 to 2.06; *n* = 499; I^2^ = 63%	**/**	**Reduction** 19 RCTs; OR 1.20, 95% CI 1.01 to 1.42, *n* = 3129; I^2^ = 2%	/	**No difference** 13 RCTs; OR 0.93, 95% CI 0.63 to 1.36, *n* = 1711; I2 = 0%	**/**
** OVULATION TRIGGERING STRATEGIES**								
**hCG type r-HCG *vs.*** **u-HCG**	**No difference** 3 RCTs; OR 1.18, 95% CI 0.50 to 2.78, *n* = 495; I^2^ = 0%	**No difference** 3 RCTs; OR 1.76, 95% CI 0.37 to 8.45, *n* = 417; I^2^ = 0%	**No difference** 7 RCTs; OR 1.15, 95% CI 0.89 to 1.49, *n* = 1136; I^2^ = 0%	**No difference** 13 RCTs; OR 1.06, 95% CI 0.87 to 1.29, *n* = 1806; I^2^ = 0%	**No difference** 7 RCTs; OR 1.15, 95% CI 0.89 to 1.49, *n* = 1136; I^2^ = 0%	**/**	**No difference** 8 RCTs; OR 0.72, 95% CI 0.41 to 1.25, *n* = 1196 I^2^ = 0%	/
**GnRH agonists**	**Reduction** 8 RCTs; OR 0.15, 95% CI 0.05 to 0.47, *n* = 989; I^2^ = 42%	**Reduction** 8 RCTs; OR 0.21, 95% CI 0.07 to 0.66, *n* = 989; I^2^ = 73%	**Reduction** 5 RCTs; OR 0.47, 95% CI 0.31 to 0.70, *n* = 532; I^2^ = 56%	/	**Reduction** 11 RCTs; OR 0.70, 95% CI 0.54 to 0.91, *n* = 198; I^2^ = 59%	/	**Increase** 11 RCTs; RR 1.74, 95% CI 1.10 to 2.75, *n* = 1198; I^2^ = 1%	/
**r-hLH**	**No difference** 2 RCTs; OR 0.83, 95%CI 0.40 to 1.70, *n* = 289; I^2^ = 6%	/	**No difference** 2 RCTs; OR 0.95, 95% CI 0.51 to 1.78, *n* = 289; I^2^ = 0%	**No difference** 2 RCTs; OR 0.94, 95% CI 0.54 to 1.64, *n* = 289; I^2^ = 0%	**No difference** 2 RCTs; OR 0.95, 95% CI 0.51 to 1.78, *n* = 289; I^2^ = 0%	/	**No difference** 2 RCTs; OR 0.95, 95% CI 0.38 to 2.40, *n* = 289; I^2^ = 0%	**No difference** 2 RCTs; MD − 1.33, 95%CI − 3.26 to 0.60, *n* = 103; I^2^ = 0%
**Elective cryopreservation**	**Reduction** 6 RCTs; OR 0.26, 95% CI 0.17 to 0.39, *n* = 4478; I^2^ = 0%	/	**No difference** 8 RCTs; OR 0.95, 95% CI 0.75 to 1.22, *n* = 4712; I^2^ = 31%	**/**	**No difference** 4 RCTs; OR 0.95, 95% CI 0.75 to 1.19, *n* = 1245; I^2^ = 31%	/	**No difference** 2 RCTs; OR 1.06, 95% CI 0.72 to 1.55, *n* = 986; I^2^ = 55%	/
**In vitro maturation of oocytes**	**No cases of OHSS** 2 RCTs; OR: not estimable; *n* = 71; I^2^: not applicable	/	/	**Increase** 2 RCTs; OR 3.10, 95% CI 1.06 to 9.00, *n* = 71; I^2^ = 0%	/	/	/	/
** OTHER TREATMENTS OR PROCEDURES**								
**Dopaminergic agonists *vs.*** **no treatment**	/	**Reduction** 10 RCTs; OR 0,32, 95% CI 0.23 to 0,44, *n* = 1202; I^2^ = 13%	**No difference** 3 RCTs; OR 0.92, 95% CI 0.63 to 1.37, *n* = 530; I^2^ = 0%	**No difference** 3 RCTs; OR 0.96, 95% CI 0.60 to 1.55, *n* = 362; I^2^ = 0%	/	/	**No difference** 2 RCTs; OR 0.66, 95% CI 0.19 to 2.28, *n* = 168; I^2^ = 0%	/
**Dopamine agonists plus co-intervention**	/	**Reduction** 4 RCTs; OR 0,48, 95% CI 0.28 to 0,84, *n* = 748; I^2^ = 40%	**No difference** 2 studies; OR 1.21, 95% CI 0.81 to 1.80, *n* = 400; I^2^ = 0%	**No difference** 4 RCTs; OR 1.11, 95% CI 0.83 to 1.49, *n* = 748; I^2^ = 0%	/	/	**No difference** 3 RCTs; OR 0.65, 95% CI 0.30 to 1.42, *n* = 548; I^2^ = 0%	/
**Diosmin**	**No difference** 1 RCT; OR 2.85, 95% CI 1.35 to 6.00, *n* = 200	/	/	**No difference** 1 RCT; OR 0.89, 95% CI 0.51 to 1.55, *n* = 200	/	/	**No difference** 1 RCT; OR 1.21, 95% CI 0.36 to 4.11, *n* = 200	/
**olume expander** **Albumin** **Hydroxyethyl starch**	**Reduction** 7 RCTs; OR 0.67, 95% CI 0.47 to 0.95, *n* = 1452; I^2^ = 69% **Reduction** 2 RCTs; OR 0.27, 95% CI 0.12 to 0.59, *n* = 272; I^2^ = 0%	/ /	/ /	/ /	/ /	**Reduction** 7 RCTs; RR 0.72, 95% CI 0.55 to 0.94, *n* = 1069; I^2^ = 42% **No difference** 1 RCTs; RR 1.20, 95% CI 0.49 to 2.93, *n* = 168	/ /	/ /
**Glucocorticoid**	**No difference** 3 RTCs; OR 1.07, 95% CI 0.60 to 1.90, *n* = 370; I^2^ = 0%	/	**No difference** 2 RCTs; OR 1.37,95% CI: 0.69 to 2.71, *n* = 366; I^2^ = 7%	**No difference** 13 RTCs; OR 1.17, 95% CI 0.95 to 1.44, *n* = 1967; I^2^ = 0%	**No difference** 3 RCTs; OR 1.19, 95% CI 0.80 to 1.76, *n* = 476; I^2^ = 0%	**/**	**No difference** 6 RCTS; OR 1.09, 95% CI 0.63 to 1.87, *n* = 821; I^2^ = 0%	/
**Luteal phase support** **hCG** **Progesterone** **GnRH agonist** (*vs*. progesterone)	**Increase** 1 RCT; OR 4.28, 95% CI 1.91 to 9.6, *n* = 387 **Reduction** 5 RCTs; OR 0.46, 95% CI 0.30 to 0.71, *n* = 1293; I^2^ = 48% **No difference** 1 RCT; OR 1.00; 95% CI 0.33 to 3.01, *n* = 300	/ / /	**Increase** 3 RCTs; OR 1.76, 95% CI 1.08 to 2.86, *n* = 527; I^2^ = 24% **No difference** 5 RCTs; OR 0.95, 95% CI 0.65 to 1.38, *n* = 833; I^2^ = 24% **Increase** 9 RCTs; OR 0.62, 95% CI 0.48 to 0.81, *n* = 2861; I^2^ = 55%	**No difference** 5 RTCs; OR 1.3, 95% CI 0.9 to 1.88, *n* = 746; I^2^ = 0% **No difference** 16 RTCs; OR 1.08, 95% CI 0.90 to 1.30 *n* = 2355; I^2^ = 0% **Increase** 8 RTCs; OR 0.66, 95% CI 0.51 to 0.85 *n* = 2435; I^2^ = 0%	**Increase** 3 RCTs; OR 1.76, 95% CI 1.08 to 2.86, *n* = 527; I^2^ = 24% **No difference** 5 RCTs; OR 0.95, 95% CI 0.65 to 1.38, *n* = 833; I^2^ = 24% **Increase** 9 RCTs; OR 0.62, 95% CI 0.48 to 0.81, *n* = 2861; I^2^ = 55%	/ / /	**No difference** 2 RTCs; OR 1.51, 95% CI 0.37 to 6.21, *n* = 140; I^2^ = 0% **No difference** 5 RTCs; OR 1.24, 95% CI 0.66 to 2.31, *n* = 832; I^2^ = 0% **No difference** 2 RTCs; OR 1.37, 95% CI 0.53 to 3.52, *n* = 240; I^2^ = 0%	/ / /
**ntensified luteal phase support**: **GnRH-a**	**No difference** 2 RCTs; RR 0.96; 95% CI 0.32–2.89, *n* = 523; l^2^ = 0%	/	**Increase** 6 RTCs; RR 1.52, 95% CI 1.20 to 1.94, *n* = 1674; l^2^ = 62%	**Increase** 11 RTCs; RR 1.21, 95% CI 1.11 to 1.33, *n* = 3038; l^2^ = 62%	**Increase** 6 RTCs; RR 1.18, 95% CI 1.06 to 1.32, *n* = 2537; l^2^ = 0%	**Increase** 6 RTCs; RR 1.36, 95% CI 1.01 to 1.82 *n* = 1164; l^2^ = 60%	/	/
**Calcium infusion**	**No difference** 2 RCTs; OR 1.83, 95% CI 0.88 to 3.81, *n* = 230; I^2^ = 81%	/	**No difference** 1 RCT; OR 1.11, 95% CI 0.66 to 1.89, *n* = 230; I^2^ = not applicable	**No difference** 2 RCTs; OR 1.00, 95% CI 0.67 to 1.49, *n* = 400; I^2^ = 0%	/	**/**	**No difference** 1 RCT; OR 1.21, 95% CI 0.27 to 1.48, *n* = 230; I^2^ = not applicable	/
**Ovarian drilling**	**No difference** 1 RCT; OR 0.27, 95% CI 0.04 to 1.69; *n*=50	/	**No difference** 1 RCT; OR 1.26, 95% CI 0.33 to 4.84, *n*=50	**No difference** 1 RCT; OR 1.20, 95% CI 0.37 to 3.86, *n*=50	/	**/**	**No difference** 1 RCT; OR 1.00, 95% CI 0.18 to 5.51, *n*=50	/

Data related to the second end-point maternal death, hospital admission and days of hospitalization are not reported since no meta-analytical data were available. In case of only one RCT, the I^2^ is not reported because not calculable/applicable

*CI* Confidence interval, *GnRH* Gonadotropin-releasing hormone, *hCG* Human chorionic gonadotropin, *IVF* In vitro fertilization, *LR* Likelihood ratio, *MD* Median difference, *n* number of subjects; OHSS; *OR* Odds ratio, *RCT* Randomized controlled trial, *RD* Risk difference, *r-hCG* Recombinant human chorionic gonadotropin, *RR* Relative risk, *SR* Systematic review, *u-hCG* Urinary human chorionic gonadotropin

### Tailoring ovarian stimulation and monitoring using OHSS risk factors

Numerous risk factors, individually or combined, have been shown to increase the overall OHSS risk. Clinical guidelines [13, 17] identify specific risk factors for recognizing OHSS high-risk patients, which may emerge before or during the IVF cycle.

#### Risk factors and predictive models

OHSS-associated risk factors are classically divided into demographic, clinical, and ovarian reserve markers. Key demographic and clinical factors include young age, polycystic ovary syndrome (PCOS) [31], ovulatory disorders [3], low body mass index (BMI) [32], history of previous OHSS [13], genetics factors [33] In terms of ovarian reserve markers, serum anti-Müllerian hormone (AMH) level above 3.36 ng/mL (with over 90% sensitivity) [34] and late follicular phase serum estradiol levels above 3,500 pg/mL [13, 35] can predict the risk of OHSS. A total antral follicle count (AFC) of 24 or higher was associated with an increased risk of moderate-to-severe OHSS [36]. On the other hand, no difference in ovarian response was detected among blood groups [37].

Various algorithms incorporating demographic/clinical and ovarian reserve data have been developed to minimize the OHSS risk, and multiple systematic reviews with meta-analyses have been conducted [38–40]. The most recent meta-analysis, comparing an ovarian reserve test-based algorithm (basal FSH, AFC and AMH) with no algorithm, found a reduction of the likelihood of moderate or severe OHSS [4 RCTs; odds ratio (OR) 0.58, 95% CI 0.34 to 1.00, *n* = 2823; I^2^ = 0%] with the use of the ovarian reserve test-based algorithm [40]. No differences in live birth / ongoing pregnancy and clinical pregnancy were observed. The CoE was low [40], and the quality assessment indicated a high-quality study.

#### Monitoring and surveillance of ovarian stimulation

Multifollicular development, elevated estradiol levels, and numerous recruited oocytes are established predictors of OHSS development [13]. Specifically, the presence of over 20 follicles during ovarian stimulation [36], retrieval of more than 24 [41] or 30 [3], oocytes, and estradiol levels exceeding 3,500 pg/mL [35] have been associated with an increased risk of OHSS. Consequently, monitoring and surveillance of ovarian stimulation may serve as a useful strategy to mitigate OHSS risk.

Two studies in the literature address this issue [42, 43]. A recent systematic review and meta-analysis reported inconclusive results regarding OHSS prevention through monitoring multifollicular development using a combination of estradiol levels and transvaginal ultrasound (TV-US) compared to TV-US alone (6 RCTs; OR 1.03, 95% CI 0.48 to 2.20, *n* = 781; I^2^ = 0%) [43]. Similarly, uncertain results were observed for the number of retrieved oocytes and pregnancy rates [43]. The certainty of evidence (CoE) was low [43], with the quality assessment indicating high quality.

### Natural cycle IVF

Natural cycle IVF involves the retrieval of an oocyte from a dominant follicle during a natural cycle, which is subsequently fertilized and cultured in vitro [44].

A systematic review with data synthesis, including only one RCT, found no evidence of a statistically significant difference in OHSS rates between natural cycle and standard IVF (1 RCT; OR 0.19, 95% CI 0.01 to 4.06, *n* = 60; I^2^ = not applicable) [45]. However, a reduction in oocyte retrieval rate was observed in natural cycle IVF, with no differences in ongoing and clinical pregnancy rates [45]. The CoE was very low [45], and the quality assessment indicated high quality.

### Pre-treatment with oral contraceptives

Pretreatment with oral contraceptive pills (OCP) has been proposed for IVF patients to enhance treatment efficacy by synchronizing the antral follicle pool prior to ovarian stimulation. Additionally, OCPs can reduce local and systemic androgen levels, especially for patients with PCOS [46].

In GnRH-ant co-treatment no effect on OHSS incidence was observed between OCP pre-treated cycles and non-pretreated cycles (2 RCTs; OR 0.98, 95% CI 0.28 to 3.40, *n* = 642; I^2^ = 0%) [47]. Live birth or ongoing pregnancy rates were lower in pretreated women, and evidence for pregnancy loss was insufficient [47]. Comparing OCP combined with the GnRH-ant protocol to the GnRH-a protocol, insufficient evidence was found to demonstrate differences in OHSS incidence (2 RCTs; OR 0.63, 95% CI 0.20 to 1.96, *n* = 290; I^2^ = 0%) or live birth or ongoing pregnancy rates. However, a reduction in miscarriage rates was observed [47]. In that study, no primary research on progestogen or estrogen pre-treatment for ovarian stimulation IVF protocols was analyzed due to a lack of data on risk of OHSS [47]. The CoE for the data was low [47], and the quality assessment indicated a high quality.

### Gonadotropin starting dose

#### Personalization

A systematic review and meta-analysis assessed the efficacy and safety of individualized gonadotropin dosing, utilizing ovarian reserve markers such as AMH, AFC and/or basal FSH [40]. The data synthesis demonstrated that personalized treatment is effective and safe for predicted high-responders, but not for predicted low- and normal-responders. In fact, a gonadotropin dosage equal to 150 UI daily or lower reduced the likelihood of moderate or severe OHSS in high-risk patients (1 RCT; OR 2.31, 95% CI 0.80 to 6.67, *n* = 521; I^2^ = not applicable) [40]. Insufficient evidence was available regarding live birth, and no difference in the clinical pregnancy was found across the treatment groups [40]. However, the evidence was scarce in terms of quality and the number of studies. The CoE was very low [40], and the quality assessment indicated a high quality.

### Mild ovarian stimulation

Mild ovarian stimulation is defined as “a procedure in which the ovaries are stimulated with gonadotropins and/or other compounds, in the intention to limit the number of oocytes obtained for IVF to fewer than seven” [48]. Three different systematic reviews with meta-analysis were identified in the literature [49–51].

The most recent meta-analysis with the highest quality confirmed a lower OHSS risk in patients receiving mild stimulation, defined as a gonadotropin administration using doses equal to or lower than 150 IU daily, compared to controls receiving a higher conventional stimulation gonadotropin dose (greater than 150 UI) in normal- (9 RCTs; RR 0.26, CI 0.14 to 0.49, *n* = 1,925; I^2^ = 0%) and hyper-responders (2 RCTs; RR 0.47, CI 0.31 to 0.72, *n* = 931; I^2^ = 0%) [51]. Conversely, no significant effect was observed in poor responders [51]. No difference was detected among normal-, poor-, and hyper-responders in terms of live-birth rates [51]. A reduction in the number of oocytes retrieved was noted in poor- and normal-responders undergoing mild stimulation compared to conventional stimulation; however, no difference between the two protocols was found in ongoing pregnancy rate [51]. The CoE was moderate [51], and the quality assessment indicated high quality.

### Drug formulation for ovarian stimulation

The first generation of gonadotropins, used in the 1970s, comprised menotropin (human menopausal gonadotropin, HMG) extracted from the urine of postmenopausal women, containing a combination of luteinizing hormone (LH) and FSH in a 1:1 ratio. Subsequently, from the early 1980s, various gonadotropins were produced, such as purified FSH (p-FSH), with less than 1 IU of LH for 75 IU of FSH, until the early 1990s, when the highly purified third-generation urinary gonadotropins (highly purified FSH, hp-FSH) were introduced, reducing the LH content to less than 0.1 IU for 75 IU of FSH. In the late 1990s, the fourth generation of gonadotropins emerged, produced through recombinant DNA technology (r-FSH), followed by the development of a recombinant LH (r-LH) formulation.

Recently, a new form of recombinant FSH was developed, corifollitropin alfa, featuring a different pharmacokinetic profile, resulting in a longer duration compared to r-FSH and requiring one injection for the first seven days of stimulation. Even more recently, follitropin delta, an r-FSH expressed only in human retinal fetal cell lines, was developed [19] along with a new recombinant human chorionic gonadotropin beta (rh-CG) [21]. To date only systematic reviews with meta-analyses comparing urinary and recombinant gonadotropins, and corifollitropin alfa and traditional gonadotropins are available.

#### Urinary vs. recombinant gonadotropins

Several systematic reviews and meta-analyses compared different gonadotropin [52–56]. The most recent high-quality study, encompassing a total of 42 trials, and 9,606 couples demonstrated no difference in the OHSS risk when comparing urinary vs. recombinant gonadotropins (32 RCTs; OR 1.18; 95% CI 0.86 to 1.61, *n* = 7,740 couples; I^2^ = 0%) [56]. Furthermore, no significant difference was observed in live birth and ongoing pregnancy rates [56]. The CoE of data was high [56], and the quality assessment indicated high quality.

#### Corifollitropin alfa

Five studies in the literature showing no significant effect of corifollitropin alfa vs. traditional gonadotropins on OHSS risk [20, 57, 58] or an increased OHSS risk [59, 60] are available. The most recent systematic review with meta-analysis reported no difference between corifollitropin alfa vs. traditional gonadotropins concerning the total risk of OHSS (5 RCTs; RR 1.15, 95% CI, 0.83 to 1.57, *n* = 3,749; I^2^ = 0%) and the risk of moderate-to-severe OHSS (4 RCTs; RR 1.17, 95% CI, 0.54 to 2.56, *n* = 3,349; I^2^ = 0% [20]. Moreover, no difference was observed regarding live birth, ongoing pregnancy, clinical pregnancy, and miscarriage rates [20]. The CoE data was not reported [20], and the quality assessment indicated moderate quality.

#### r-LH

LH supplementation is effective in improving pregnancy rates in patients with severe LH deficiency [61]. Even if with scarce scientific evidence, it is also used in the clinical practice in presence of hypo-response to r-hFSH and in patients with serum LH levels deeply suppressed. Proofs-of-concept and experimental data also suggest that r-hLH supplementation may reduce OHSS risk, as LH appears to suppress the small antral follicles during gonadotropin ovarian stimulation [62].

A systematic review of RCTs with meta-analysis analyzed the effects on OHSS incidence of r-LH combined with r-FSH in ovarian stimulation in comparison with r-FSH alone, demonstrating no significant effect (6 RCTs; OR 0.38, 95% CI 0.14 to 1.01, *n* = 2,178; I^2^ = 10%) [63]. No significant difference in the live birth rate and miscarriage rate was found, although the ongoing pregnancy rate was reduced [63]. The CoE data was low [63], and the quality assessment indicated high quality.

### Regimens of ovarian stimulation

#### Clomiphene citrate (CC) and/or letrozole

The incorporation of CC and/or letrozole with gonadotropins has been proposed to mitigate OHSS risk through a mechanism not entirely understood. CC stimulates endogenous FSH and LH secretion by competing for estrogen receptors at the hypothalamic level, potentially leading to the initial growth of fewer dominant follicles during subsequent ovarian stimulation with exogenous gonadotropins [64]. Letrozole, an aromatase inhibitor, increases endogenous FSH and LH release and exerts negative feedback on the pituitary by reducing circulating estradiol levels through inhibition of androgen aromatization into estrogens in ovarian granulosa cells, without impacting peripheral tissue estrogen receptors [64, 65].

A study incorporating data on CC or letrozole administration showed a reduction in the OHSS risk for normal- and poor-responder patients in both GnRH-a and GnRH-ant co-treated cycles (5 RCTs; OR 0.21, 95% CI 0.11 to 0.41, *n* = 1067; I^2^ = 0%) [66]. Concurrently, no significant differences were observed in live birth and clinical pregnancy rates, although a reduction in the number of oocytes retrieved was noted in the general unselected population [66]. The CoE data was low [66], and the quality assessment indicated moderate quality.

Available studies [67, 68] on CC corroborated the beneficial effect of CC on OHSS risk. The most recent systematic review with meta-analysis revealed a significant reduction in the risk of OHSS in CC-treated patients compared to a standard ovarian stimulation (4 RCTs; OR 0.15, 95% CI, 0.07 to 0.32 *n* = 1,251; I^2^ = 0%) [68]. Both GnRH-a and GnRH-ant IVF cycles were included [68]. Despite a significant reduction in oocyte retrieval in CC cycles, no differences were detected between the two groups regarding clinical pregnancy and live birth rates. The CoE was moderate [68], and the quality assessment indicated moderate quality.

A significant reduction in OHSS risk was reported in letrozole-treated patients compared to standard ovarian stimulation protocols with GnRH-a or GnRH-ant co-treatment (1 RCT; OR 0. 1.95% CI, 0. 0 to 0. 6 *n* = 94; I^2^ = not applicable) [68]. Due to data scarcity and low evidence level, no conclusions could be drawn concerning other assessed outcomes [68]. The CoE was low [68], and the quality assessment indicated moderate quality.

#### Metformin

Several mechanisms have been proposed to explain metformin’s beneficial effects on OHSS risk reduction [69]. These include insulin-sensitizing actions with reductions in insulin and IGF-1 level, anti-inflammatory effects with reductions in serum VEGF levels, and anti-androgenic effect with reductions in intraovarian androgen levels and restoration of a normal FSH sensitivity of the granulosa cell [69]. Numerous systematic reviews and meta-analyses were evaluated [70–77].

The selected meta-analysis study demonstrated that metformin supplementation in GnRH-a IVF cycles significantly reduces the OHSS risk (9 RCTs; RR 0.40, 95% CI 0.26 to 0.60, *n* = 898; I^2^ = 13%) [77]. However, these results were not replicated in GnRH-ant IVF cycles (2 RCTs; RR 0.97, 95% CI 0.32 to 2.98, *n* = 193; I^2^ = 26%) [77]. In long GnRH-a down-regulation protocols, metformin improved clinical pregnancy rate, although no effect on live birth rates was observed [77]. Conversely, in GnRH-ant protocols, metformin appeared to reduce in live birth rates [77]. The CoE was low [77], and the quality assessment indicated high quality.

#### Melatonin

Melatonin, a free radical scavenger that stimulates antioxidant enzymes to protect cells from oxidative stress [78], has been studied to improve oocyte quality in IVF programs.

A systematic review of RCTs, which included only one study, demonstrated that melatonin supplementation did not influence the OHSS risk (1 RCT; RR 1.01, 95% CI 0.33 to 3.08, *n* = 358; I^2^ = not applicable) [79]. No significant differences were observed in clinical pregnancy and miscarriage rates [79]. The CoE was very low [79], and the quality assessment indicated high quality.

### Coasting

Coasting, an OHSS prevention strategy involving gonadotropin suspension and delaying hCG administration until a significant reduction in serum estradiol level is achieved [80].

A meta-analysis showed that coasting effectively reduces OHSS risk (2 RCTs; OR 0.11, 95% CI 0.05 to 0.24, *n* = 207; I^2^ = 0%) [81]. However, insufficient evidence was available to assess the procedure’s efficacy in terms of live birth, clinical pregnancy, and miscarriage rate [81]. The CoE was low [81], and the quality assessment indicated high quality.

### Strategies for controlling the LH surge

Inhibition of the LH surge is crucial for optimizing safety and efficacy in IVF cycles. GnRH agonists, GnRH antagonists, or progestogens are currently used for this purpose.

#### GnRH analogues

The two primary and effective approaches for LH surge prevention in IVF cycles involve pituitary desensitization via prolonged daily administration of a GnRH-a or immediate LH secretion blocking with a GnRH-ant. Several studies evaluating their efficacy and safety in the general population [82–84], in PCOS patients [84–89], in normal responders [90, 91], and poor responders [84] have been intercepted.

A systematic review with meta-analysis, which included all RCTs comparing the efficacy and safety of GnRH-ant to the long-course GnRH-a protocol without restriction for the type of IVF population, demonstrated a significantly lower incidence of any grade of OHSS in GnRH-ant cycles compared to GnRH-a cycles (36 RCTs; OR 0.61, 95% C 0.51 to 0.72, *n* = 7,944; I^2^ = 31%) [83]. No significant difference was seen in live birth, ongoing pregnancy rates, clinical pregnancy rates and miscarriage [83]. The CoE was moderate [83], and the quality assessment indicated high quality.

##### General population

In a systematic review with meta-analysis of RCTs including general IVF patients (unselected for ovarian response or other characteristics), the incidence of any grade of OHSS was significantly lower in GnRH-ant cycles compared to long GnRH-a down-regulation cycles (22 RCTs; OR 0.63, 95% CI 0.50 to 0.81, *n* = 5,598; I^2^ = 0%) [84]. A reduction in ongoing pregnancy rates and clinical pregnancy rates was detected, without a significant effect on live birth rates [84]. However, the type of GnRH-ant administration (flexible or fixed) influenced the efficacy data because no evidence of a difference in any clinical outcome was observed between GnRH-ant and GnRH-a when a fixed antagonist protocol was used with and without OCP pre-treatment [84]. The CoE of data was reported as moderate [84]. The quality assessment showed a high quality.

##### PCOS

In PCOS patients [89], a reduction in the risk of OHSS was observed in those treated with GnRH-ant compared to those receiving a long-course GnRH-a protocol (9 RCTs; OR 0.65, 95% C 0.52 to 0.82, *n* = 1,114; I^2^ = 0%). However, no differences in live birth rate, ongoing pregnancy rate, clinical pregnancy rate and miscarriage rate were observed [89]. The CoE was very low [89], and the quality assessment indicated high quality. Unfortunately, a sub-analysis for GnRH-ant protocols was not performed [89].

A previous high-quality systematic review of RCTs with meta-analysis confirmed that the use of a GnRH-ant was effective in reducing OHSS risk in PCOS patients, both when used as a fixed (3 RCTs; RR 0.94, 95% CI 0.63 to 1.40, *n* = 434; I^2^ = 0%) and flexible (7 RCTs; RR 1.02, 95% CI 0.79 to 1.36, *n* = 814; I^2^ = 0%) protocol [84]. No differences in ongoing pregnancy rate, live birth, and clinical pregnancy rate were observed in that sub-analysis [84]. In all primary studies, OCP was administered before ovarian stimulation. The CoE of data was moderate [84], and the quality assessment indicated high quality.

##### Normal responders

A study analyzing the efficacy of GnRH-ant in presumed normal responders, i.e., IVF patients with a normal ovarian reserve, found a significantly lower risk of OHSS using the GnRH-ant protocol compared to the GnRH-a long-protocol was seen (21 RCTs; OR 0.69, 95% CI 0.57 to 0.83, *n* = 5,763; I^2^ = 15%) [91]. No differences in live birth rate, ongoing pregnancy rate, clinical pregnancy rate and miscarriage was observed between two protocols, even though a lower oocyte number was retrieved in GnRH-ant protocols [91]. No sub-analysis for GnRH-ant protocols (fixed and flexible) was performed [91]. The CoE of data was not reported [91], and the quality assessment indicated high quality.

##### Poor responders

A systematic review with meta-analysis of RCTs comparing the GnRH-ant protocol with the long-course GnRH-a protocol and including poor responders did not find any primary study with OHSS data (6 RCTs; *n* = 780) [84]. No difference in live birth, ongoing pregnancy, clinical pregnancy rate was detected [84]. The CoE of data was moderate [84], and the quality assessment indicated high quality.

#### Progestin-primed ovarian stimulation (PPOS)

PPOS involves the oral administration of exogenous progestogens, such as medroxyprogesterone acetate or dydrogesterone, from the early follicular phase. This approach prevents the activation and transmission phases of estradiol-induced LH surges in IVF cycles [92] and is combined with a “freeze-all” strategy.

Only one systematic review with meta-analysis of RCTs compared the PPOS protocol with other protocols, such as GnRH-ant, GnRH-a, and natural cycle [93]. The data were sub-analyzed according to different ovarian reserves, including poor responders, normal responders, and PCOS patients [93]. The PPOS protocol was associated with a reduced risk of OHSS (6 RCTs; OR 0.52, 95% CI 0.36 to 0.75, *n* = 240; I^2^ = 0%) [93]. No differences in live birth/ongoing pregnancy, clinical pregnancy rate, and the number of retrieved oocytes were observed [93]. Data sub-analysis demonstrated no difference between the PPOS protocol and other specific protocol subgroups, except for the comparison with the GnRH-ant protocol in OHSS incidence (4 RCTs; RR 0.54, 95% CI 0.37 to 0.79, *n* = 901; I^2^ = 0%). The CoE of data was low [93], and the quality assessment indicated low quality.

### Ovulation triggering strategies

OHSS is a postovulatory syndrome resulting from spontaneous or iatrogenic ovulation induction. Therefore, specific ovulation induction strategies in IVF cycles are crucial for OHSS prevention.

#### hCG

The hCG trigger is currently the gold standard trigger concept in normal and poor responder patients undergoing autologous fresh embryo transfer [17].

#### Type of hCG

The only systematic review with meta-analysis of RCTs aimed to compare the different types of hCG demonstrated no significant differences between recombinant hCG (r-hCG) and urinary hCG (u-hCG) concerning OHSS risk (3 RCTs; OR 1.18, 95% CI 0.50 to 2.78, *n* = 495; I^2^ = 0%) [94]. Moreover, no difference in live birth, clinical pregnancy, ongoing pregnancy, and miscarriage rates was seen [94]. The CoE of data was low [94], and the quality assessment indicated high quality.

#### GnRH-a

GnRH-a administration is effective for triggering final oocyte maturation in IVF cycles downregulated with a GnRH-ant. Several systematic reviews of RCTs with meta-analysis were identified [94–96]. The most recent and highest quality systematic review with meta-analysis of RCTs [96] demonstrated the efficacy of the GnRH-a trigger compared to an hCG trigger for final oocyte maturation in terms of lowering the OHSS risk (8 RCTs; OR 0.15, 95% CI 0.05 to 0.47, *n* = 989; I^2^ = 42%) [96]. However, a reduction in live birth and ongoing pregnancy rates, and an increase in early miscarriage rates were observed in fresh autologous transfer cycles after GnRH-a triggering (without hCG rescue) compared to the standard hCG trigger [96]. The CoE of data was moderate [96], and the quality assessment indicated high quality.

#### r-LH

r-LH possesses the same biological and pharmacokinetic characteristics as human pituitary LH, making it effective for inducing final follicular maturation with a significant reduction in OHSS when a single dose of up to 30,000 IU is used for triggering [97].

Only one systematic review with meta-analysis of RCTs was intercepted [94]. Meta-analytic data found no significant difference in OHSS risk in IVF patients who received r-LH compared to patients who received u-hCG (2 RCTs; OR 0.83, 95% CI 0.40 to 1.70, *n* = 289; I^2^ = 6%) [94]. No differences were observed for live birth/ongoing pregnancy rate, clinical pregnancy rate, miscarriage rate, and the number of oocytes retrieved between treatment [94]. The CoE of data was very low [94], and the quality assessment showed high quality.

### Elective cryopreservation

One of the first strategies used to prevent/reduce the OHSS risk was the freezing of the embryos. The cryopreservation of all embryos avoiding the transfer may reduce the hCG production and stimulus from initial pregnancy and, consequently, the early OHSS form [15]. Elective cryopreservation, also known as the “freeze-all strategy”, is a strategy consisting of planning an IVF cycle in which all embryos are frozen and transferred in subsequent frozen-thaw embryo cycles, also known as “cycle segmentation” [98].

Two systematic reviews with meta-analysis of RCTs were intercepted [99, 100]. The first [99] is an updating of previous studies and analyzed the effectiveness of embryo freezing in comparison with human intra-venous albumin infusion or with fresh embryo transfer. Only two RCTs were identified (one for each comparison). No difference was found in all the outcomes (including OHSS) showing insufficient evidence to support routine embryo freezing for reducing the OHSS risk. These results have been incorporated in most recent meta-analysis [100].

Individual meta-analysis reported that elective cryopreservation is associated with a reduction in OHSS risk (6 RCTs; OR 0.26, 95% CI 0.17 to 0.39, *n* = 4,478; I^2^ = 0%) compared to conventional embryo transfer in woman scheduled for IVF, which consists of fresh embryo transfer followed by the subsequent transfer of supernumerary embryos [100]. No difference was found in live birth, cumulative pregnancy and miscarriage rates between elective cryopreservation and the conventional strategy [100]. The CoE of data was low [100], and the quality assessment indicated high quality.

### In vitro maturation (IVM) of oocytes

In vitro maturation (IVM) refers to the maturation in culture of immature oocytes, which may or may not have been exposed to short periods of gonadotropin stimulation. After retrieval, the final stages of maturation are completed in vitro during culture [101]. Our systematic search detected only systematic review with meta-analysis of RCTs comparing IVM vs. IVF or ICSI in PCOS patients. No OHSS case was detected in IVM patients (2 RCTs; OR: not estimable, *n* = 71, I^2^ = not applicable) [102]. A higher clinical pregnancy rate was observed in IVM compared to IVF [102]. Other data were not available because both RCTs were published as abstracts. The CoE of data was very low [102], and the quality assessment indicated high quality.

### Other treatments or procedures

#### Dopaminergic agonists

Dopaminergic agonists, such as cabergoline, quinagolide, and bromocriptine, bind to dopaminergic receptors, promoting endocytosis of the VEGF receptor and subsequently reducing neovascularization and vascular permeability [103].

Several systematic reviews with meta-analyses have been conducted [104–106]. The most recent analysis demonstrated that dopaminergic agonists effectively prevent moderate-severe OHSS compared to no treatment and/or placebo (10 RCTs; OR 0.32, 95% CI 0.23 to 0.44, *n* = 1,202; I^2^ = 13%) [106]. Furthermore, their efficacy was significantly superior compared to other co-interventions, such as coasting, albumin, prednisolone, calcium infusion, etc. (4 RCTs; OR 0.48, 95% CI 0.28 to 0.84, *n* = 748; I^2^ = 40%) [106]. No difference was observed regarding live birth, clinical pregnancy, and miscarriage rates [106]. The CoE of data was moderate [106], and the quality assessment indicated high quality.

#### Diosmin

Diosmin, a natural flavonoid commonly used to treat chronic venous diseases, exerts various pharmacological effects, including anti-inflammatory and antioxidant actions [107].

Only one systematic review with meta-analysis was identified [106]. It included only one RCT and showed no difference in OHSS risk between diosmin and cabergoline (1 RCT; OR 2.85, 95% CI 1.35 to 6.00, *n* = 200; I^2^ = not applicable) [106]. No differences in clinical pregnancy and miscarriage rates were also observed between patients treated with diosmin and those who received cabergoline. The CoE of data was very low [106], and the quality assessment showed high quality.

#### Volume expanders

Various volume expanders, including albumin, hydroxyethyl starch (HES), mannitol, polygeline, and dextran, have been used over the years to prevent OHSS with inconclusive results [108, 109]. Several mechanisms have been proposed to explain the potential effect of volume expanders on OHSS prevention, including increased intravascular volume, osmotic pressure, reduction in platelet aggregation and reduction in blood coagulation [110].

A single systematic review and meta-analysis of RCTs has been identified [111]. Meta-analytic data demonstrated that intravenous administration of human albumin at the time of oocyte retrieval reduced the incidence of moderate-to-severe OHSS compared to no treatment or placebo in OHSS high-risk patients (7 RCTs; OR 0.67, 95% CI 0.47 to 0.95, *n* = 1,452; I^2^ = 69%) [111]. However, a reduction in pregnancy rate was observed [111]. HES administration also reduced OHSS risk compared to placebo (2 RCTs; OR 0.27, 95% CI 0.12 to 0.59, *n* = 272; I^2^ = 0%) but did not affect the pregnancy rate [111]. The CoE of data was very low for both albumin and HES administration [111], and the quality assessment indicated high quality.

#### Glucocorticoid administration

Glucocorticoids have been suggested to improve folliculogenesis and pregnancy rates and enhance the intrauterine environment by functioning as immunomodulators, reducing the number and activity of natural uterine killer (NK) cells, normalizing the endometrial cytokine expression profile, and suppressing endometrial inflammation [112].

As a result, supplementation has been proposed during ovarian stimulation [113] and the peri-implantation period [114]. Regarding the peri-implantation period [115], no difference in OHSS was found compared to placebo/ no treatment (3 RTCs; OR 1.07, 95% CI 0.60 to 1.90, *n* = 370; I^2^ = 0%). No difference was detected in live birth, ongoing pregnancy, clinical pregnancy, and miscarriage rates. The CoE data was very low [114]. and the quality assessment was high. On the other hand, concerning glucocorticoid supplementation for ovarian stimulation, no studies reported OHSS or side effects [113].

#### Traditional luteal phase support

The luteal phase of all stimulated cycles is disrupted, as supraphysiological steroid levels (estradiol and progesterone) during the early-mid luteal phase exert a negative feed-back on the hypothalamic-pituitary axis, reducing LH secretion during the early luteal phase [115]. Consequently, luteal phase support is critical in bridging the gap between the disappearance of the exogenous hCG administered for ovulation trigger and the initiation of endogenous hCG secretion by the trophoblast of the implant [116].

In the systematic review with meta-analysis on RCTs intercepted the administration of hCG for luteal phase support after the classic hCG ovulation trigger significantly increases the risk of OHSS compared to no treatment (1 RCT; OR 4.28, 95% CI 1.91 to 9.6, *n* = 387; I^2^ = not applicable) [117]. However, a beneficial statistical trend of hCG vs. no treatment in live birth and ongoing pregnancies was found. No difference in clinical pregnancy and miscarriage was also found [117]. Progesterone administration resulted in a lower risk of OHSS, when compared to hCG (5 RCTs; OR 0.46, 95% CI 0.30 to 0.71, *n* = 1,293; I^2^ = 48%), and no difference in live birth, ongoing pregnancy, clinical pregnancy, and miscarriage rate was detected [117]. No difference was observed in OHSS rate when exploring the use of the GnRH-a for 3 days after embryo transfer in association with progesterone compared with progesterone alone (1 RCT; OR 1.00; 95% CI 0.33 to 3.01, *n* = 300; I^2^ = not applicable) [117]. Higher live birth, ongoing pregnancy and clinical pregnancy rates were detected. No difference in miscarriage rate was found [117]. The CoE data was low for all previous comparisons [117]. and the assessment of quality indicated high quality.

#### Intensified luteal phase support

##### GnRH-a

GnRH-a administration was used as intensive luteal phase support after GnRH-a trigger [118, 119].

A recent systematic review of RCTs with meta-analysis detected in our search showed no difference in OHSS risk in patients who received, GnRH agonist as luteal phase support compared to progesterone (2 RCTs; RR 0.96; 95% CI 0.32 to 2.89, *n* = 523; I^2^ = 0%) [120]. Improved live birth, clinical pregnancy, ongoing pregnancy, and pregnancy rates were detected [120]. The CoE data was not reported [120]. and the assessment of quality indicated high quality.

#### Calcium infusion

Increased serum calcium levels may inhibit cyclic adenosine monophosphate (cAMP)-stimulated renin secretion, decrease the production of angiotensin-converting enzyme II synthesis, and reduce VEGF expression in human luteinized granulosa cells [121, 122]. Based on this rationale, the intravenous administration of calcium on the day of oocyte retrieval and days 1, 2, and 3 after oocyte retrieval was studied as an intervention to decrease the risk of OHSS. Only one systematic review with meta-analysis of RCTs was intercepted. This meta-analysis compared dopamine agonists to calcium infusion and detected no difference in OHSS incidence between the two groups (2 RCTs; OR 1.83, 95% CI 0.88 to 3.81, *n* = 230; I^2^ = 81%) [106]. No difference was detected in a live birth, clinical pregnancy, and miscarriage rates [106]. The CoE data was very low [106], and the quality assessment indicated high quality.

#### Ovarian drilling

Ovarian drilling is a surgical laparoscopic or vaginal technique performed in patients with PCOS and consisting in the destruction of ovarian tissue. The result is endocrine modifications characterized by the reduction in androgens and LH levels and the increase in FSH levels leading to both reduced follicular androgenic dominance in favor of estrogenic dominance [123] and the reconstitution of the physiological pituitary ovary feedback mechanisms, promoting follicular recruitment and ovulation, and minimizing the risks of OHSS [124].

A systematic review of RCTs with meta-analysis demonstrated no effect of LOD in infertile patients with PCOS who IVF cycles (1 RCT; OR 0.27, 95% CI 0.04 to 1.69; *n *= 50; I^2^ = not applicable) [125]. No difference in live birth, clinical pregnancy, ongoing pregnancy, and miscarriage rates was seen [125]. The CoE of data was very low [125], and the quality assessment indicated high quality.

## Discussion

This is the first systematic umbrella review that aims to comprehensively identify and critically analyze the most effective evidence-based interventions for preventing or reducing the incidence and severity of OHSS in patients undergoing IVF. Systematic reviews with meta-analysis were intercepted using the PICO model [26] and in accordance with the PRIOR guidelines [25], that overcome methodological challenges of the previous overviews of reviews using pragmatic approach.

We confirm the efficacy of several interventions in reducing the incidence and severity of OHSS. The use of GnRH-ant, with or without GnRH-a triggering (with embryo freezing) remains the best strategy to prevent OHSS, even if a reduction in clinical pregnancy rates was also found in general/unselected IVF populations. In “freeze all” IVF cycles, PPOS protocol seems to be also effective in reducing OHSS risk. Additionally, other interventions may also be clinically beneficial for high-risk OHSS patients who undergo GnRH-a down-regulation, as they appear to reduce the risk of OHSS with minimal or no minimal impact on reproductive outcomes. Such interventions include lower doses of exogenous gonadotropins for ovarian stimulation, metformin coadministration, and dopamine agonists. On the other hand, many interventions, including coasting and CC administration, have a negative impact on reproductive outcomes, and cannot be suggested. Intriguingly, limited data exists on potential interventions for preventing OHSS and reducing its severity in GnRH-ant cycles, aside from GnRH trigger.

Our systematic analysis identified a total of 37 interventions for OHSS prevention analyzed in 28 systematic reviews of RCTs with meta-analyses. We included 27 systematic reviews of RCTs with meta-analyses. The AMSTAR-2 methodological quality assessment of the studies was high, moderate, and low for 23, 2, and 3 studies, respectively. The CoE, reported for each specific intervention and in each specific situation/population, was high in only one case, while it was moderate to very low for the others.

Six years ago, another previous review of reviews was published [24]. It summarized evidence from 27 Cochrane systematic reviews on interventions for prevention or treatment of moderate, severe, and overall OHSS in patients undergoing IVF [24]. The systematic reviews analyzed were generally of high quality, albeit only evidence of moderate quality was identified. Specifically, the use of metformin before and during IVF cycles, the use of GnRH-ant protocols and GnRH-a triggering in oocyte donors or ‘freeze-all’ programs were effective [24]. In comparison with previous review of Cochrane reviews [24], current umbrella review includes 13 new interventions and 4 updated Cochrane reviews including one non-Cochrane review [24]. Thus, our data significantly update and expand the knowledge about the potential interventions for reducing the risk of OHSS in IVF patients.

We decided to exclude systematic reviews with network meta-analyses from our protocol design, as they provide mixed evidence from direct and indirect comparisons and are based on the assumption of transitivity among comparisons [126]; importantly, their scientific and clinical results remain under debate [27]. However, our systematic research identified two well-performed recent network meta-analyses of RCTs [127, 128]. Marino et al. [127] reported that algorithm-based strategies were more effective in reducing OHSS compared to experience-based treatment and standard gonadotropin dosing. No significant differences were observed in live birth and clinical pregnancy rates between strategies [127] Wu et al. [128] demonstrated a significant effect of HES and cabergoline in reducing the incidence of moderate-to-severe OHSS compared to placebo or blank controls. Letrozole, aspirin, albumin, metformin, and quinagolide did not prevent moderate-to-severe OHSS [128]. All interventions had a grade of evidence ranging from moderate to high and were considered safe in terms of reproductive outcomes [128].

Current review has strengths and limitations. The strengths include an extensive literature search of specific potential interventions affecting the incidence of OHSS, adherence to the PICO model [26] and thorough quality assessment following PRIOR guidelines [25] to detect potential biases (AMSTAR-2). The main limitations are the low quality of evidence in the available studies and overlapping interventions in many meta-analyses introducing confounders. In many cases, little populations were studied with few events reported, which may not coincide with common clinical evidence. For example, no effect of the use of natural cycles was detected, even if it is obvious that mono-follicle development is associate with a risk clearly lower in OHSS risk when compared to multiple follicular development. Several systematic reviews with meta-analyses are outdated. To this regard, we did not consider the publication period as a restriction criterion and included the most recent studies with the highest evidence hierarchy in the final analysis. In addition, crucial secondary endpoints, such as the incidence of maternal deaths, and the incidence and length of the hospital admission for OHSS, were not included and analyzed in the included studies. Regarding the effect of the interventions on the reproductive outcomes, the live birth and pregnancy rates were generally reported per fresh ET in the original studies, even ifs the cumulative rate of live births / pregnancy per stimulation cycle should be a more effective measure to assess the intervention safety. Finally, several other promising interventions, such as follicotropin delta [129–131] or kisspeptin [132], were not analyzed and discussed because not yet supported by meta-analytical evidence (see Table 2).

Several considerations arise from reviewing the available literature. First, much data concerning GnRH-ant cycles have been published in recent years, while evidence-based data about GnRH-a cycles are dated. This is of particular interest as a large number of GnRH-a cycles are still performed worldwide [133] and recent clinical trials in new gonadotropin formulations [19–21] seem to reintroduce the use of GnRH-a also in scientific and academic settings. Although, the GnRH-ant protocol should be preferred in the presumed high-risk OHSS patients [17], identifying high-risk patients remains an unsolved issue, and a formal consensus defining a patient as a “hyper-responder” is currently lacking. Moreover, OHSS should be considered in all women undergoing ovarian stimulation for fertility treatment, as the condition is largely unpredictable and genetic predisposition plays a crucial role [22]. Testing the efficacy of various interventions without an adequate underlying scientific background, which is surprising considering the substantial human and economic resources required for clinical trials. We emphasize the need to follow the standard clinical trial phases; however, we encountered numerous phase 3 clinical trials without adequate preceding phase 1 or 2 studies. Third, we did not find further studies aiming to optimize the dose, timing, or other characteristics of treatments/interventions, even for clinical studies showing a moderate effect of specific interventions on OHSS risk. Lastly, despite inconsistencies in available evidence-based data, our systematic review identified recent studies with conflicting findings. Similarly, recent systematic reviews of non-randomized studies obtained mixed results on letrozole [128, 133], while others confirmed efficacy of metformin in non-obese PCOS patients [71]. These studies may confuse readers and affect the clinical management of IVF patients.

In conclusion, present comprehensive umbrella review identified specific evidence-based interventions to prevent or reduce the incidence and severity of OHSS in IVF patients. Specifically, in suspected high-risk patients the use of GnRH-ant should be preferred, and the GnRH-a triggering with embryo freezing considered in case of persistent high-risk. PPOS protocol may be a valid option in case of elective embryo transfer or for cancer patients in the context of fertility preservation or for donor patients. In patients who undergo GnRH-a down-regulated cycles, the use of mild stimulation seems to be a safe approach, and metformin coadministration during ovarian stimulation may be effective to reduce the risk such as dopamine agonists administration after oocyte triggering. Even if not based on solid evidence but according to the common sense, the embryo freezing should be considered in all cases of persistent high-risk for OHSS.

However, our review also highlighted a scientific gap regarding interventions in both GnRH-ant and GnRH-a co-treated IVF cycles. As OHSS remains a significant clinical challenge, further well-designed studies are warranted to provide updated, reliable, and consistent evidence on prevention and management strategies. Before the use of genomics in reproductive medicine will be able to select patients at risk for OHSS, these advancements will ultimately help clinicians to tailor personalized treatment plans to reduce OHSS risk and improve patient safety and reproductive outcomes in assisted reproductive technology.

## Data Availability

The data underlying this article will be shared on reasonable request to the corresponding author.

## Abbreviations

AFC: Antral follicle count
AMH: Anti-Mullerian Hormone
AMSTAR-2: Assessing the Methodological Quality of Systematic Reviews 2
ART: Assisted reproductive technology
ASRM: American Society for Reproductive Medicine
BMI: Body mass index
cAMP: Cyclic adenosine monophosphate
CC: Clomiphene citrate
CoE: Certainty of evidence
FSH: Follicle-stimulating hormone
GnRH: Gonadotropin releasing hormone
GnRH-a: Gonadotropin releasing hormone agonist
GnRH-ant: Gonadotropin releasing hormone antagonist
hCG: Human chorionic gonadotropin
HES: Hydroxyethyl starch
HMG: Human menopausal gonadotropin
hp-FSH: Highly purified follicle stimulating hormone
ICSI: Intracytoplasmic sperm injection
IGF: Insulin-like growth factor
IL: Interleukin
IVF: In vitro fertilization
IVM: In vitro maturation
LH: Luteinizing hormone
NK: Natural uterine killer
OCP: Oral contraceptive pills
OHSS: Ovarian hyperstimulation syndrome
OR: Odds ratio
PCOS: Polycystic ovary syndrome
PPOS: Progestin-primed ovarian stimulation
p-FSH: Purified follicle stimulating hormone
PICO: Population, Intervention, Comparison, Outcome
PRIOR: Preferred Reporting Items for Overviews of Reviews
RCT: Randomized controlled trial
r-FSH: Recombinant follicle stimulating hormone
r-hCG: Recombinant human chorionic gonadotropin
r-LH: Recombinant luteinizing hormone
RR: Relative risk
TGF: Transforming growth factor
TV-US: Transvaginal ultrasound
u-hCG: Urinary human chorionic gonadotropin
US: United States
VEGF: Vascular endothelial growth factor
